# *S*electing the *o*ptimal positio*n* of CDK4/6 *i*nhibitors in hormone receptor-positive *a*dvanced breast cancer – the SONIA study: study protocol for a randomized controlled trial

**DOI:** 10.1186/s12885-018-4978-1

**Published:** 2018-11-20

**Authors:** A. van Ommen-Nijhof, I. R. Konings, C. J. J. van Zeijl, C. A. Uyl-de Groot, V. van der Noort, A. Jager, G. S. Sonke, On behalf of the SONIA study steering committee, Sylvia Luykx-de Bakker, Hiltje de Graaf, Wouter Dercksen, Aafke Honkoop, Alex Imholz, Quirine van Rossum-Schornagel, Ester Siemerink, Inge Baas, Rianne Oosterkamp, Carolien Schröder, Judith Kroep, Annebeth Haringhuizen, Cathrien Tromp-van Driel, Frans Erdkamp, Paul de Jong, Jolien Tol, Yes van de Wouw, Joan Heijns, Anne-Marie van Riel, Caroline Mandigers, Marga Schrieks

**Affiliations:** 1 The Netherlands Cancer Institute, PO Box 90203, 1006 BE Amsterdam, The Netherlands; 2 Amsterdam UMC, location VUmc, PO Box 7057, 1007 MB Amsterdam, The Netherlands; 3 Erasmus School of Health Policy & Management / Institute for Medical Technology Assessment, Erasmus University Rotterdam, PO Box 1738, 3000 DR Rotterdam, The Netherlands; 4 Erasmus MC Cancer Institute, PO Box 5201, 3008 AE Rotterdam, The Netherlands

**Keywords:** Advanced breast cancer, Metastatic breast cancer, Palbociclib, Ribociclib, Abemaciclib, CDK4/6 inhibitors, Non-steroidal aromatase inhibitors, Fulvestrant

## Abstract

**Background:**

Combining cyclin-dependent kinases 4 and 6 (CDK4/6) inhibitors with endocrine therapy is an effective strategy to improve progression-free survival in hormone receptor-positive (HR+), human epidermal growth factor receptor 2 (HER2)-negative advanced breast cancer. There is a lack of comparative data to help clinicians decide if CDK4/6 inhibitors can best be added to first- or second-line endocrine therapy. Improvement in median progression-free survival in first-line studies is larger than in second-line studies, but CDK4/6 inhibitors have not consistently shown to improve overall survival or quality of life. They do come with added toxicity and costs, and many patients have lasting disease remission on endocrine therapy alone. No subgroup has been identified to select patients who are most likely to benefit from the addition of CDK4/6 inhibition in any line of treatment. Altogether, these factors make that the optimal strategy for using CDK4/6 inhibitors in clinical practice is unknown.

**Methods:**

The SONIA study is an investigator-initiated, multicenter, randomized phase III study in patients with HR+/HER2-negative advanced breast cancer. Patients are randomly assigned to receive either strategy A (first-line treatment with a non-steroidal aromatase inhibitor combined with CDK4/6 inhibition, followed on progression by fulvestrant) or strategy B (first-line treatment with a non-steroidal aromatase inhibitor, followed on progression by fulvestrant combined with CDK4/6 inhibition). The primary objective is to test whether strategy A is more effective than strategy B. The primary endpoint is time from randomization to second objective progression (PFS2). Secondary endpoints include overall survival, safety, quality of life, and cost-effectiveness. Five-hundred seventy-four events yield 89% power to show that strategy A has statistically significant, clinically meaningful superior PFS2 (according to ESMO-MCBS) in a log-rank test at the two-sided 95% confidence level. Given an accrual period of 42 months and an additional 18 months follow-up, inclusion of 1050 evaluable patients is required.

**Discussion:**

This study design represents daily clinical practice, and the results will aid clinicians in deciding when adding CDK4/6 inhibitors to endocrine therapy will benefit their patients most. Additional biomarker analyses may help to optimize patient selection.

**Trial registration:**

http://clinicaltrials.gov: NCT03425838 (8 February 2018). EudraCT-number: 2017–002334-23 (29 September 2017).

**Electronic supplementary material:**

The online version of this article (10.1186/s12885-018-4978-1) contains supplementary material, which is available to authorized users.

## Background

Hormone receptor-positive (HR+), human epidermal growth factor receptor 2 (HER2)-negative breast cancer is the most common subset of breast cancer [1]. About one-third of all HR+/HER2-negative patients, initially diagnosed with early stage disease, experience disease recurrence [2]. As a result, the HR+/HER2-negative subset is responsible for the majority of breast cancer related deaths.

For decades, the mainstay treatment of HR+ advanced breast cancer (ABC) has been sequential endocrine therapies targeting the estrogen receptor-signaling pathway. Although endocrine therapies may lead to durable disease control, the majority of patients progress during endocrine therapy (acquired resistance) and there is also a proportion of patients that fails to respond to initial therapy (de novo resistance) [3].

In an effort to overcome endocrine resistance, treatments with novel molecular targets have been developed, including cyclin-dependent kinase 4 and 6 (CDK4/6) inhibitors. CDK4/6 inhibitors prevent cell cycle progression from G1 into S phase [4]. Several (pre)clinical studies have shown efficacy of combining CDK4/6 inhibitors with endocrine therapies in HR+/HER2-negative ABC [5–9]. Currently, three CDK4/6 inhibitors have been FDA- and EMA-approved: palbociclib, ribociclib, and abemaciclib.

In first-line setting, adding CDK4/6 inhibitors to aromatase inhibitors leads to approximately doubling of progression-free survival (PFS). The PALOMA-2 phase III trial randomized patients between either letrozole and palbociclib or letrozole and placebo. Median PFS in the palbociclib-group was 24.8 months versus 14.5 months in the control group (hazard ratio (HR) 0.58 (95% confidence interval [CI]: 0.46–0.72)) [8]. The MONALEESA-2 trial with ribociclib resulted in a median PFS in the combination group (ribociclib with letrozole) of 25.3 months versus 16.0 months in the control group (placebo with letrozole) with an HR of 0.57 (95% confidence interval [CI]: 0.46–0.70) [10, 12]. In the MONARCH-3 trial, abemaciclib was used. Median PFS was not reached in the combination group (abemaciclib with either letrozole or anastrozole) versus 14.7 months in the control group (placebo with either letrozole or anastrozole), with an HR of 0.54 (95% confidence interval [CI]: 0.41–0.72) [9].

Combining CDK4/6 inhibitors with fulvestrant proved to be an effective strategy in second and subsequent treatment lines. In the PALOMA-3 phase III trial patients whose disease had progressed after one or more previous lines of therapy were treated with either fulvestrant and palbociclib or fulvestrant and placebo. The PFS in the combination group (palbociclib with fulvestrant) was 9.5 months versus 4.6 months in the control group (placebo with fulvestrant) with an HR of 0.46 (95% confidence interval [CI]: 0.36–0.59) [5]. The MONALEESA-3 study compared fulvestrant with or without ribociclib in patients who were treatment naïve or had received up to one line of prior endocrine therapy. Median PFS increased from 12.8 months in the fulvestrant + placebo group to 20.5 months in the fulvestrant + ribociclib group (HR 0.59; 95% confidence interval [CI]: 0.48-0.73) [13]. Combining abemaciclib with fulvestrant as second-line treatment in the MONARCH-2 trial resulted in a median PFS of 16.4 months versus 9.3 months in the control group (placebo with fulvestrant) with an HR of 0.55 (95% confidence interval [CI]: 0.45–0.68) [11]. Different from PALOMA-3, only one prior line of endocrine therapy was allowed and no chemotherapy for metastatic disease was allowed. This probably accounts for the differences in median PFS between both studies. The hazard ratios, however, are very similar.

The above-mentioned studies have led to the firm belief that combining CDK4/6 inhibitors with endocrine therapy leads to an improved PFS in HR+/HER2-negative advanced breast cancer. Remarkably, no benefit in overall survival (OS) could be demonstrated so far. The only available mature OS data at this point arise from the preceding phase II PALOMA-1 trial. In this study, OS was very similar with and without palbociclib [14]. A recently published update of the PALOMA-3 data did not show a statistically significant overall survival benefit for the addition of palbociclib to fulvestrant in the entire trial group [15].

Combining CDK4/6 inhibition with endocrine therapy is associated with increased toxicity compared to endocrine therapy alone [5, 8–11]. Almost 70% of patients experience CTCAE grade 3–4 toxicity with the combination compared to 20% with endocrine therapy alone. Patients treated with combination therapy primarily experience bone marrow suppression, but may also experience fatigue, diarrhea, liver dysfunction, and QTc prolongation. As a result, the use of CDK4/6 inhibitors requires more frequent hospital visits and controls. The average duration of use of CDK4/6 inhibitors is substantially longer in first-line compared to second- or subsequent lines (approximately 25 months and 9–16 months, respectively). This means patients are subjected to potential side effects and more frequent hospital visits for a longer period of time when CDK4/6 inhibitors are used as a first-line treatment. This might be part of the reason why despite the clear benefit in PFS, adding a CDK4/6 inhibitor to endocrine therapy to first-line treatment does not clearly result in improved quality of life (QoL). Health-related QoL, as assessed by validated questionnaires, did not differ between treatment arms in the PALOMA-2 and MONALEESA-2 trial [16, 17]. The effect of CDK4/6 inhibition is consistent across a wide range of subgroups based on clinical and pathological characteristics. As a consequence, no predictive biomarkers exist to select patients who are most likely to benefit from the addition of CDK4/6 inhibition.

In summary, it is currently not known which treatment strategy for deploying CDK4/6 inhibitors will benefit patients most. Adding CDK4/6 inhibitors in first- compared to second-line endocrine treatment may provide longer PFS benefit but is associated with longer use of the drugs, resulting in more toxicity and costs, without clear benefit on OS and QoL [18]. The aim of this project is to evaluate whether the sequence of an AI plus CDK4/6 inhibition in first-line followed by fulvestrant in second-line (strategy A) is superior to the sequence of an AI in first-line followed by fulvestrant plus CDK4/6 inhibition in second-line (strategy B).

## Methods/design

**Fig. 1:**
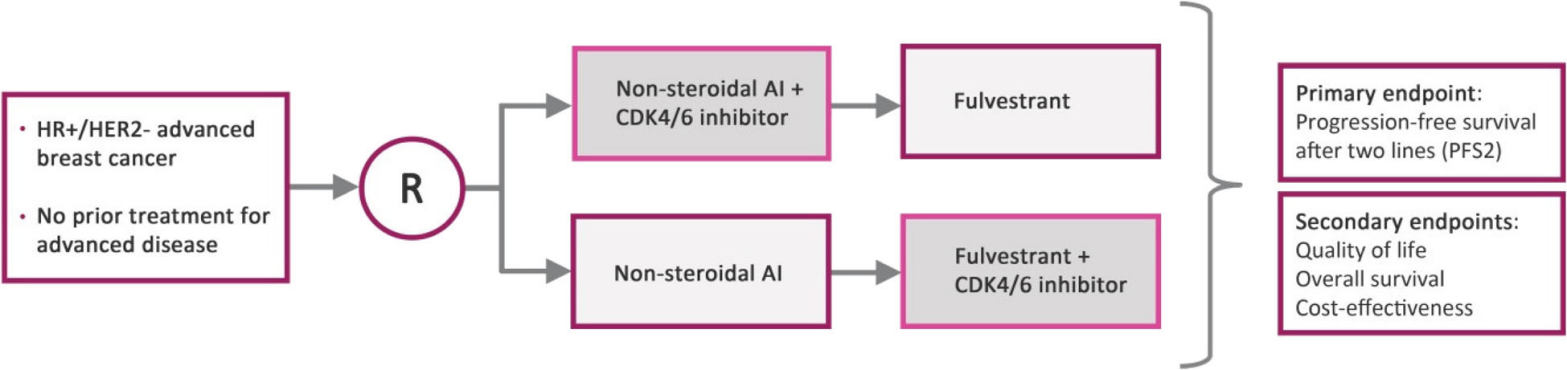
Schematic overview of study design. Non-steroidal AI: non-steroidal aromatase-inhibitor, either letrozole or anastrozole (at the discretion of the treating physician). CDK4/6 inhibitor: type of CDK4/6 inhibitor according to physician’s choice and reimbursement policies

The SONIA study is an investigator-initiated nationwide, multicenter, randomized phase III study in women with HR+/HER2-negative advanced breast cancer who have not received any prior systemic anti-cancer therapy for advanced disease. Patients are randomly assigned to receive either strategy A (first-line treatment with a non-steroidal aromatase inhibitor combined with CDK4/6 inhibition, followed on progression by fulvestrant) or strategy B (first-line treatment with a non-steroidal aromatase inhibitor, followed on progression by fulvestrant combined with CDK4/6 inhibition). A schematic overview of the study design is shown in Fig. 1.

### Objectives

The primary objective is to test whether CDK4/6 inhibition added to the non-steroidal aromatase inhibitor in first-line treatment (strategy A) is more effective than addition of CDK4/6 inhibition to fulvestrant in second-line treatment (strategy B). The primary endpoint is progression-free survival after two lines (PFS2), defined as time from randomization until objective disease progression, symptomatic deterioration, or initiation of a new therapeutic agent on second-line treatment, death, or progression during a break in initial therapy and without further therapy within one month, whichever occurs first. When a patient dies while on first-line treatment or is ineligible to continue to second-line endocrine treatment according to protocol, her PFS2 equals her PFS1. Secondary objectives include comparisons of OS, QoL, safety and tolerability, objective response rate (ORR) and cost-effectiveness. Additional biomarker analyses are planned, involving pharmacokinetics and pharmacogenetics analyses as well as baseline, on-treatment and on-progression tumor biopsies, liquid biopsies and nuclear imaging; patients are required to provide additional informed consent for these procedures.

### Inclusion criteria

Eligible patients have histologically or cytologically proven diagnosis of adenocarcinoma of the breast with evidence of locoregional recurrent or metastatic disease and will be candidates to receive non-steroidal AIs as first-line treatment for their advanced disease. Patients are not candidates for curative therapies and may not have received any prior systemic anticancer therapies for metastatic disease other than a maximum of 28 days of palliative endocrine treatment. (Neo)adjuvant aromatase inhibitor use is allowed, provided that the disease did not progress while on or within 12 months from ending treatment. Patients must have measurable or evaluable disease as per RECIST 1.1 [19]. Bone lesions visible on bone scan or fludeoxyglucose positron emission tomography (FDG PET) should be confirmed by computed tomography (CT) or magnetic resonance imaging (MRI) at baseline. The patient or a legal representative must be able and willing to give informed consent.

### Exclusion criteria

Patients are excluded from this study in case of advanced, symptomatic, visceral spread with the risk of life-threatening complications in the short term and in case of known active uncontrolled or symptomatic metastases of the central nervous system (including leptomeningeal disease). Patients who are currently being treated with strong cytochrome P450 3A4 (CYP3A4) inducers or inhibitors or were previously treated with CDK4/6 inhibitors, are not amenable for study participation. Other conditions excluding a patient from participating are other malignancies (except those that are not believed to influence the patient’s prognosis and do not require any further treatment), prolonged QTc time (> 480 ms), bowel disease interfering with the uptake of oral medication, or any other medical condition that interferes with study procedures or compliance.

### Interventions

Patients will be randomized in a 1:1 ratio to receive either strategy A or strategy B. Patients randomized to strategy A will receive first line treatment with letrozole 2.5 mg once daily or anastrozole 1 mg once daily continuously in combination with either palbociclib 125 mg once daily or ribociclib 600 mg once daily on day 1–21 of every 28-day cycle. Second-line treatment will consist of fulvestrant 500 mg on day 1, day 15 and day 29 of the first month and monthly thereafter. Patients randomized to strategy B will receive letrozole 2.5 mg once daily or anastrozole 1 mg once daily continuously as first-line treatment. Second-line treatment will consist of fulvestrant 500 mg on day 1, day 15 and day 29 of the first month and monthly thereafter in combination with palbociclib 125 mg once daily on day 1–21 of every 28-day cycle. The proposed study design will be amended upon reimbursement to allow ribociclib also in strategy B and abemaciclib as third alternative CDK4/6 inhibitor. Patients will be stratified by site of disease (visceral versus non-visceral), prior endocrine treatment in (neo) adjuvant setting (yes versus no), hospital, and type of CDK4/6 inhibitor. All pre- and perimenopausal women will also receive ovarian ablation or suppression. Patients will continue to receive the assigned study medication until objective disease progression, symptomatic deterioration, or unacceptable toxicity (at the discretion of the treating physician), death, or withdrawal of consent, whichever occurs first. Patients will be screened at baseline for eligibility, and after signing informed consent, baseline measurements will be obtained. Subsequently, patients will be followed up according to their treatment group. Patients randomized to strategy A will undergo laboratory evaluation after 2, 4, 6, and 8 weeks and monthly thereafter. Patients randomized to strategy B will undergo laboratory evaluation three-monthly during first-line treatment. During second-line treatment, the follow-up schedule for patients in group A will be identical to that of patients in group B during first-line treatment and vice versa. In both treatment arms, response assessment and safety evaluation will be performed every three months by means of imaging, laboratory evaluation, evaluation of ECOG performance status, physical examination, and recording of adverse events and concomitant medication. Questionnaires regarding QoL and cost-effectiveness will be administered at several prespecified time points in the study. A table with all study-related activities can be found in Additional file 1.

### Dose modifications

Every effort should be made to administer study treatment on the planned dose and schedule. However, in the event of significant treatment-related toxicity, administration of CDK4/6 inhibitors may need to be adjusted. Dose adjustment is not permitted for non-steroidal AIs, fulvestrant or LHRH agonists. Depending on the nature of the toxicity observed, treatment interruption of CDK4/6 inhibitors may be required. Treatment with letrozole/anastrozole or fulvestrant will continue as pre-planned. For more information about dose modifications, see Additional file 2.

### Concomitant treatment

Standard therapies for pre-existing medical conditions, medical and/or surgical complications, and palliation are allowed during study participation. Palliative radiotherapy is allowed during the study, although it is recommended to initiate radiotherapy before study initiation if possible. Caution is advised on theoretical grounds for any surgical procedures during the study. The appropriate interval of time between surgery and CDK4/6 inhibitors required to minimize the risk of impaired wound healing and bleeding has not been determined. Medications not allowed during study participation include other anticancer agents, strong CYP3A4 inhibitors or inducers, hormone replacement therapy and hormonal contraception.

### Statistics: Sample size and power calculations

The primary outcome for this study is PFS2, defined as time from randomization to second objective progression. The threshold for a clinically relevant difference in PFS2 is set according to the European Society for Medical Oncology - Magnitude of Clinical Benefit Scale (ESMO-MCBS) [20]. The maximal preliminary score for efficacy based on PFS is 3 out of 4, with a higher score indicating more clinical benefit (see Table 1). The preliminary scores can be upgraded or downgraded depending on data on toxicity and quality of life to achieve the final score.

**Table 1:** preliminary magnitude of clinical benefit grade based on PFS

Grade 3	HR ≤0.65 and gain ≥3 months
Grade 2	HR ≤0.65 but gain < 3 months
Grade 1	HR > 0.65

*PFS* progression-free survival, *HR* hazard ratio (thresholds refer to the lower extreme of the 95% confidence interval)

Based on the PALOMA-studies, we estimated strategy A to show a median PFS2 of 31 months and strategy B to show a median PFS2 of 28 months, with a corresponding hazard ratio of 0.85. For strategy A to be preferable according to ESMO-MCBS, a statistically significant difference in PFS of at least three months and a lower limit of the 95% confidence interval ≤ 0.65 for the corresponding hazard ratio is required. In the power calculation we implemented these minimum requirements by multiplying the estimated hazards in the palbociclib + letrozole group (PALOMA-2) and in the palbociclib + fulvestrant group (PALOMA-3) by 0.88 while multiplying the estimated hazards in the letrozole single agent group (PALOMA-2) and fulvestrant single agent group (PALOMA-3) by 1/0.88. This modification of the actual hazards yields expected median PFS2 times of 32.6 and 27.4 months in the strategy A and strategy B, respectively.

Based on 10,000 simulated trials with the modified hazards we conclude that inclusion of 500 patients per arm in a period of 42 months and an additional 18 months follow-up will yield an expected number of 574 events and 89% power to show that strategy A has statistically significant superior PFS2 in a log-rank test at the two-sided 95% confidence level. The corresponding hazard ratio for strategy A: strategy B is 0.765 (95% CI 0.648–0.902) and the probability of meeting the above-mentioned ESMO-MCBS criteria is 52%. If the study fails to detect superiority, non-inferiority will be tested next. According to the closed test principle, hierarchical testing of superiority and non-inferiority does not affect sample size or power of the analyses.

### Statistics: Analysis

#### Efficacy

The difference in PFS2 (primary endpoint) will be estimated using the intention-to-treat (ITT) population in a Cox proportional hazards model accounting for all stratification factors. An adjusted hazard ratio with a symmetric 1-α confidence interval will be produced.

Secondary objectives include comparisons of OS, ORR, QoL, safety and tolerability and cost-effectiveness. A stratified log-rank test (using the same stratification factors as for the PFS analysis) will be used to compare OS between the treatment strategies. The ORR on each randomized treatment strategy will be estimated by dividing the number of patients with objective response (either complete response (CR) or partial response (PR) according to RECIST 1.1) by the number of patients randomized to the respective treatment strategy (“response rate”). A 95% CI for the response rates will be provided. Response rate comparisons between the two treatment strategies as randomized will be assessed using Cochran-Mantel-Haenszel (CMH) test with the same stratification factors as for the PFS analysis. Analyses of ORR will be performed on the ITT population. Health related QoL will be analyzed using patient reported outcomes of the disease specific FACT-B and generic EQ-5D-5 L questionnaires [21, 22] (https://euroqol.org/eq-5d-instruments/eq-5d-5l-about/). The FACT-B and EQ-5D scoring guidelines will be used to compute FACT module specific scores, overall FACT-B quality of life scores, and EQ-5D utility scores. The cost and outcomes of both arms will be assessed by Mann Whitney test. Cost-effectiveness will be determined by comparing costs and effects of both treatment strategies by means of the Institute for Medical Technology Assessment Resource Use Questionnaire Borstkanker (iMTA-RUQ-B). This questionnaire was developed by iMTA by combining questions from the iMTA Productivity Cost Questionnaire (iPCQ), the iMTA Medical Consumption Questionnaire (iMCQ) and the iMTA Valuation of Informal Care Questionnaire (iVICQ) (https://www.imta.nl/questionnaires/) [23]. Quality adjusted life years (QALY) will be computed by multiplying life-years with the observed utility scores during those life years. As a societal perspective will be taken into account all relevant direct and indirect costs will be measured. In this respect, the friction cost method will be used for estimating the societal costs of productivity losses.

#### Compliance

It is conceivable that patients upon first progression will not proceed to the second phase of the study but instead start a different treatment. While this will not prevent us to analyze their data (for these patients the PFS2 will be considered equal to the PFS1), a large fraction of patients deviating from protocol will nevertheless devaluate the results of the study, as it then answers a different question than was originally asked. Based on simulations, the change in the observed hazard ratio comparing PFS2 in both treatment arms can exceed 0.05 when > 15% of patients are non-compliant in their second line treatment. Therefore, we will do an interim analysis for compliance, where we stop inclusion into the study early when more than 15% of patients that had progression in first-line did not go on to second-line according to protocol. This evaluation will be carried out at the moment that 100 patients in arm A had a PFS1 event. If the study is stopped at that moment, all patients already in the study (expected to be around 670) will be followed until PFS2 and analyzed according to protocol.

#### Safety

The As Treated (AT) population will be the primary population for safety evaluation. Summaries of adverse events and other safety parameters will be provided as appropriate. The study will use a Data Safety and Monitoring Board (DSMB). The DSMB will be responsible for ongoing monitoring of the safety of patients in the study. The DSMB will make a recommendation as to whether or not the study should continue based on ongoing reviews of safety data.

### Legislation and logistics

The SONIA study was approved by the accredited Medical Ethics Committee of the Netherlands Cancer Institute – Antoni van Leeuwenhoek (METC AVL) on September 29th, 2017. The study will be conducted in accordance with legal and regulatory requirements, as well as the general principles set forth in the International Ethical Guidelines for Biomedical Research Involving Human Subjects, Guidelines for good Clinical Practice (GCP), and the Declaration of Helsinki. A total of 74 hospitals in the Netherlands will participate. Patients will be informed about the study and asked to participate by their treating physician.

A centralized internet/telephone registration system (ALEA) is used for randomization. All data will be recorded by qualified data managers in an electronic case report form (eCRF), which is managed by “Borstkanker Onderzoekgroep” (BOOG), the sponsor of this study. On site monitoring will take place conform the guideline of the “Nederlandse Federatie van Universitaire Medisch Centra” (NFU) called “Kwaliteitsborging van mensgebonden onderzoek 2012” by the appointed monitor.

## Discussion

The SONIA study aims to determine the optimal position of addition of CDK4/6 inhibitors to standard endocrine treatment in patients with advanced, HR+/HER2-negative breast cancer by evaluating its efficacy, safety, quality of life and cost-effectiveness in first-line compared to second-line treatment. The study is designed to reflect daily clinical practice and is in line with current treatment guidelines [24, 25].

PFS2 was selected as a primary outcome, since it will be readily available and is the best available surrogate for OS [26]. Because all patients in our study will have received CDK4/6 inhibitors (either in first- or in second-line) when they reach the end of the study, our results will not be confounded by post-trial therapy with the investigational drug. Secondary outcomes include OS (historically the most unambiguous efficacy marker [27]), QoL (an important patient-reported outcome measure) and cost-effectiveness. In addition, we are planning to perform biomarker analyses, which may contribute to current knowledge on determining which patients will or will not benefit from the addition of CDK4/6 inhibition to standard endocrine treatment.

Trial status: we are currently recruiting patients.

## Additional files

Additional file 1: Summary of study-related activities (DOCX 308 kb)

Additional file 2: Summary of recommendations regarding dose modifications (DOCX 19 kb)
