# Supplementary material for: Selecting the optimal position of CDK4/6 inhibitors in hormone receptor-positive advanced breast cancer – the SONIA study: study protocol for a randomized controlled trial
Source: BMC Cancer. 2018 Nov 20;18:1146. doi: 10.1186/s12885-018-4978-1 (PMC6247672; doi:10.1186/s12885-018-4978-1)
Supplement: Supplementary file 1 — Summary of study-related activities (DOCX 308 kb) [file 12885_2018_4978_MOESM1_ESM.docx]

###### Additional file 2

**Summary of study-related activities**


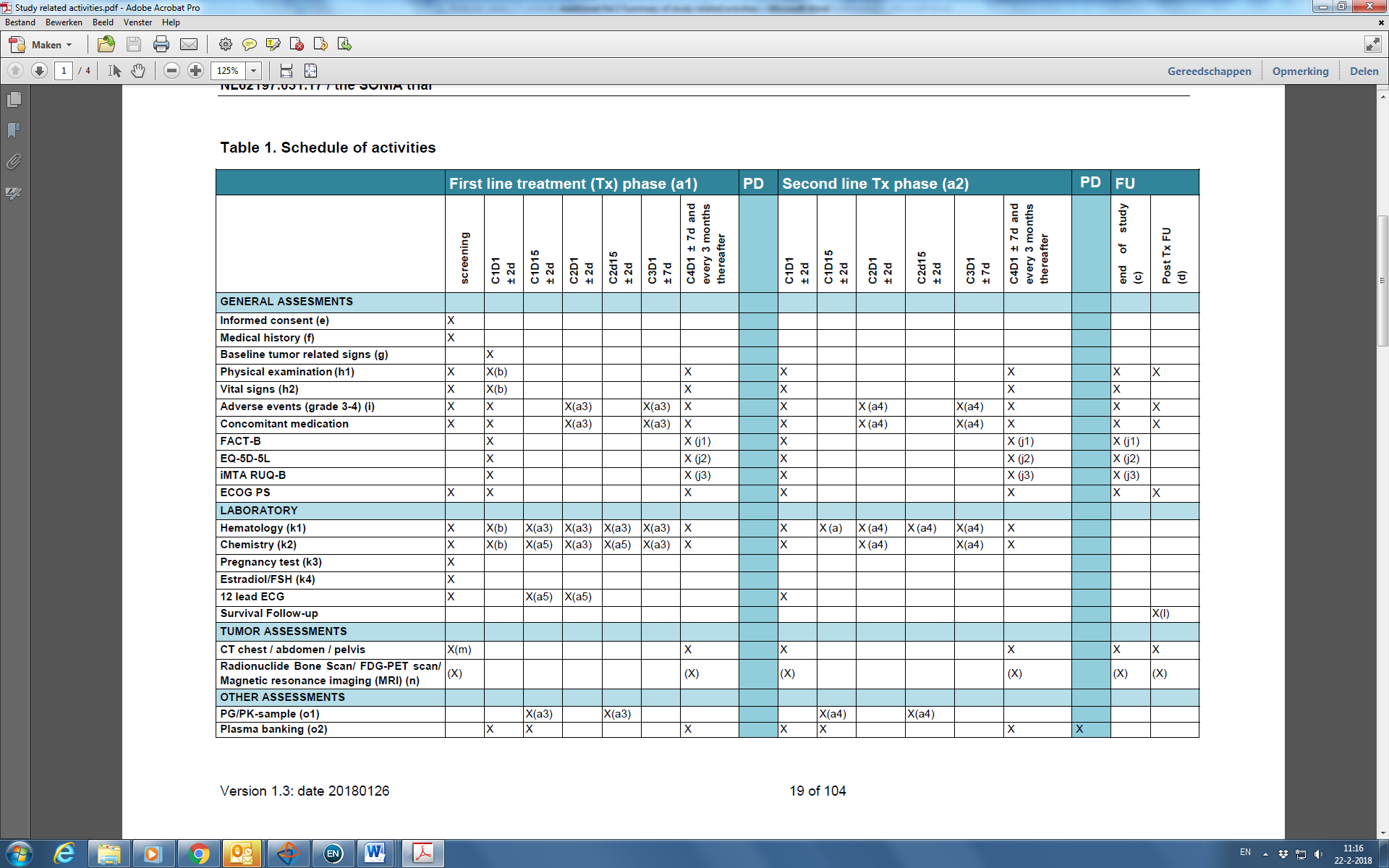


**a1.** **First line treatment phase:** A cycle is defined as 28 days; Cycle 1 Day 1 is defined as C1D1

Strategy A: starting on C1D1 with non-steroidal aromatase inhibitor (+ LHRH agonist if pre- or perimenopausal) plus CDK4/6 inhibitor

Strategy B: starting on C1D1 with non-steroidal aromatase inhibitor (+ LHRH agonist if pre- or perimenopausal)

The first line treatment phase continues as long as the patient is tolerating anticancer drugs well and does not show progressive disease at response evaluation.

**a2. Second line treatment phase:** A cycle is defined as 28 days; Cycle 1 Day 1 (C1D1) is the first cycle after progression and starting point for second line therapy

Strategy A: starting on C1D1 with fulvestrant (+ LHRH agonist if pre- or perimenopausal)

Strategy B: starting on C1D1 with fulvestrant (+ LHRH agonist if pre- or perimenopausal) plus palbociclib

The second line treatment phase continues as long as the patient is tolerating anticancer drugs and does not show progressive disease at response evaluation.

**a3.** Strategy A only

**a4.** Strategy B only

**a5.** For ribociclib only (safety evaluation)

**b.** **Cycle 1/Day 1 (C1D1):** chemistry, hematology, and physical examination is not required if screening assessments were performed <7 days prior to randomization.

**c. End of study (participation/withdrawal):** visit to be performed as soon as possible but within four weeks from the last dose of anticancer drug and prior to initiation of any new anticancer therapy.

**d.** **Post Tx (treatment) follow-up:** patients who discontinue anticancer treatment within this study during first or second line for any reason other than objective disease progression or death will continue to have tumor assessments performed every 12 weeks after start of the latest active treatment course until documented progression or onset of new anticancer therapy.

**e. Informed consent:** Informed consent must be obtained prior to any protocol required assessments being performed

**f**. **Medical (including oncological) history:** including information on all prior anticancer treatments.

**g**. **Baseline tumor related signs (and/or symptoms):** baseline tumor related signs and/or symptoms will be recorded at the C1D1 visit prior to initiating first line treatment and then reported as adverse events during the trial if they worsen in severity to grade 3 or 4

**h1**. **Physical examination:** full physical examination including an examination of all major body systems which may be performed by a physician, registered nurse or other qualified health care provider.

**h2**. **Vital signs:** height (at screening only), blood pressure, pulse rate (at C1D1 of each line) and weight (at screening and every 12 weeks, ± 7 days)which may be performed by a physician, registered nurse or other qualified health care provider.

**i.** **Adverse Events (AEs):** serious adverse events (SAEs) grade 3 or grade 4 must be reported from the time the patient provides informed consent through and including 28 calendar days after the last administration of the study drug. SAEs occurring after the active reporting period has ended should be reported if the investigator becomes aware of them; at a minimum, all grade 3 or 4 SAEs that the investigator believes have at least a reasonable possibility of being related to study drug are to be reported to the Sponsor. All grade 3 or 4 AEs and all SAEs should be recorded on the CRF from the first dose of study treatment through last patient visit.

**j1. FACT-B:** the FACT-B questionnaire will be administered at baseline (screening or C1D1) and after 3, 6 and 12 months on first line treatment and at start of second line treatment and after 3, 6 and 12 months on second line treatment and at progression after second line treatment

**j2.** **EQ-5D-5L:** the EQ-5D-5L questionnaire will be administered at baseline (screening or C1D1) and after 3, 6 and 12 months on first line treatment and at start of second line treatment and after 3, 6 and 12 months on second line treatment and at progression after second line treatment.

**j3. iMTA RUQ-B:** the iMTA RUQ-B questionnaire will be administered at baseline and after 6 months on first line treatment and at start of second line treatment, after 6 months on second line treatment and at progression after second line treatment.

**k1. Laboratory tests: hematology** includes hemoglobin, WBC, absolute neutrophils, platelet count.

**k2. Laboratory tests: chemistry** includes AST/ALT, alkaline phosphatase, GGT, total bilirubin, sodium, potassium, total calcium, serum creatinine, and albumin**.**

**k3. Laboratory tests**: **pregnancy test** (serum or urine) at screening only for women with childbearing potential.

**k4. Laboratory tests**: **serum estradiol and follicle stimulating hormone (FSH)** levels are analyzed at screening to confirm postmenopausal status of women <60y with amenorrhea for at least 12 consecutive months.

**l. Survival follow-up**: for patients who discontinue study treatment due to objective disease progression after second line treatment, or after withdrawing consent, survival data (i.e. patient status along with start, stop and type of new anticancer therapy) will be collected yearly.

m. CT chest/ abdomen/pelvis: FDG-PET + a diagnostic CT is also allowed at screening however, diagnostic CT scan of chest/abdomen/pelvis should be used for response evaluations during the study. Radiographic tumor assessments that were performed as routine procedures before signing the informed consent form, but within 28 days prior to randomization, do not need to be repeated and may be used as baseline assessments. Tumor assessments are performed every 12 weeks (± 7 days, after 5 years on first or second line treatment ± 14 days) until disease progression during first and second line treatment.

**n.** **Radionuclide bone scan / FDG-PET / MRI:**  additional whole body imaging with FDG-PET or bone scintigraphy is necessary at screening if no bone lesion is visible on CT scan. Any suspicious abnormality identified on nuclear imaging at baseline as only site of disease must be confirmed by MRI or CT. In case of bone only disease not visible on CT scan, follow-up should be performed by means of nuclear imaging (either radionuclide bone scan or FDG-PET; the imaging modality that was chosen at screening, should also be used in follow-up). New lesions or flare of known lesions should be confirmed by other imaging modalities. CT scans in this specific situation are not mandatory but can be performed at the discretion of the physician.
