# Supplementary material for: Selecting the optimal position of CDK4/6 inhibitors in hormone receptor-positive advanced breast cancer – the SONIA study: study protocol for a randomized controlled trial
Source: BMC Cancer. 2018 Nov 20;18:1146. doi: 10.1186/s12885-018-4978-1 (PMC6247672; doi:10.1186/s12885-018-4978-1)
Supplement: Supplementary file 2 — Summary of recommendations regarding dose modifications (DOCX 19 kb) [file 12885_2018_4978_MOESM2_ESM.docx]

###### Additional file 2

###### Summary of recommendations regarding dose modifications

###### Table S1a. Available Dose Levels of palbociclib

| **Dose Level** | **Palbociclib for 3 out of 4 weeks (3/1 schedule)** |
| --- | --- |
| Starting dose | 125 mg/d |
| First dose reduction | 100 mg/d |
| Second dose reduction | 75 mg/d |

###### Table S1b. Available Dose levels of ribociclib

| **Dose Level** | **Ribociclib for 3 out of 4 weeks (3/1 schedule)** |
| --- | --- |
| Starting dose | 600 mg/d |
| First dose reduction | 400 mg/d |
| Second dose reduction | 200 mg/d |

###### Table S2a. Management advice for hematological toxicity

*Note: retreatment criteria must be met within 3 weeks of dose interruption*

| **Toxicity** | **Management** |
| --- | --- |
| **Grade 3**  - Hb < 4.9 mmol/L or transfusion required  - T <50.0 - 25.0 x 10^9^  - ANC <1.0 - 0.5 x 10^9^ | Day 1 of cycle: withhold CDK 4/6 inhibitor, repeat complete blood count monitoring within 1 week. When recovered to Grade ≤2, start cycle at the same dose.  Day 15 of cycle: continue CDK 4/6 inhibitor at current dose to complete cycle.  Restart at next lower dose level in case of prolonged (>1 week) spontaneous recovery, necessity of transfusion or recurrent Grade 3 event in subsequent cycles. |
| **Grade 4**  - Anemia with life-threatening consequences; urgent intervention indicated  - T <25 x 10^9^  - ANC <0.5 x 10^9^ | Withhold CDK 4/6 inhibitor until recovery to Grade ≤2.  Resume at next lower dose level. |
| **Grade 3 neutropenia with fever**  - ANC <1.0 x 10^9^ with a single temperature of >38.3⁰C or a sustained temperature of >=38⁰C for more than one  hour | Withhold CDK 4/6 inhibitor until recovery of ANC to Grade ≤2 and resolution of fever.  Resume at next lower dose level. |
| **Grade 4 neutropenia with fever**  - Febrile neutropenia with life-threatening consequences; urgent intervention indicated | Withhold CDK 4/6 inhibitor at least until recovery of ANC to Grade ≤2 and resolution of fever.  It is at the discretion of the treating physician to resume at the next lower dose level or discontinue altogether. |

Hb = haemoglobin; T = thrombocyte count; ANC = absolute neutrophil count

###### Table S2b. Management advice for hepatobiliary toxicity

*Note: retreatment criteria must be met within 3 weeks of dose interruption*

| **Toxicity** | **Management** |
| --- | --- |
| **Grade ≥2 increased bilirubin (with the exception of Gilbert’s syndrome)**  (>1.5 x ULN) | Withhold CDK 4/6 inhibitor until recovery to Grade <2.  Resume at next lower dose. |

ULN = upper limit of normal

###### Table S2c. Management advice for prolonged QTc (for ribociclib only!)

*Note: retreatment criteria must be met within 3 weeks of dose interruption*

| **Toxicity** | **Management** |
| --- | --- |
| **QTc >480 msec - ≤500 msec** | Withhold ribociclib, repeat ECG weekly.  If QTc prolongation resolves to <481 msec: resume treatment at same dose level.  If QTc ≥481 msec recurs: withhold ribociclib until QTc resolves to <481 msecr. Resume at next lower dose level. |
| **QTc >500 msec** | Withhold ribociclib, repeat ECG weekly.  If QTc prolongation resolves to <481 msec: resume treatment at next lower dose level.  If QTc prolongation >500 msec or greater than 60 msec change from baseline occurs in combination with torsade de pointes or polymorphic ventricular tachycardia or signs/symptoms of serious arrhythmia, discontinue ribociclib. |

###### Table S2d. Management advice for all other toxicity

*Note: retreatment criteria must be met within 3 weeks of dose interruption*

| **Toxicity** | **Management** |
| --- | --- |
| **Grade 2** | In case of Grade 2 lasting longer than 3 weeks (except alopecia): withhold CDK 4/6 inhibitor until recovery to Grade ≤1 or baseline. Resume at same dose level. |
| **Grade 3 or 4** | Withhold CDK 4/6inhibitor until symptoms resolve to:  - Baseline  - Grade ≤1;  - Grade ≤2 (if not considered a safety risk for the patient)  Resume at the next lower dose. |
